# Xingnaojing Injection Protects against Cerebral Ischemia Reperfusion Injury via PI3K/Akt-Mediated eNOS Phosphorylation

**DOI:** 10.1155/2018/2361046

**Published:** 2018-08-08

**Authors:** Yue-Ming Zhang, Xiao-Yu Qu, Jing-Hui Zhai, Li-Na Tao, Huan Gao, Yan-Qing Song, Si-Xi Zhang

**Affiliations:** Department of Pharmacy, The First Hospital of Jilin University, Changchun, Jilin 130021, China

## Abstract

Xingnaojing (XNJ) injection, derived from traditional Chinese medicine formulation, has a protective effect against stroke, but the underlying mechanism is unclear, which severely limited its clinical application. This research aims to elucidate the role and mechanism of XNJ in reducing cerebral ischemic reperfusion (I/R) injury. Rats received 2 h cerebral ischemia followed by reperfusion of 24 h and were intraperitoneally given 5, 10, or 15 ml/kg XNJ 24 h before ischemia and at the onset of reperfusion, respectively. TTC staining, HE staining, and neurological score were implied to evaluate the effectiveness of XNJ. The protein expressions of PI3K/Akt and eNOS signaling were measured. Experiments were further performed in human brain microvascular endothelial cells (HBMECs) to investigate the protective mechanisms of XNJ. HBMECs were subjected to 3 h oxygen and glucose deprivation following 24 h of reoxygenation (OGD) to mimic cerebral I/R in vitro. PI3K inhibitor LY294002 was added with or without the preconditioning of XNJ. Multiple methods including western blot, immunofluorescence, DAPI staining, JC-1, and flow cytometry were carried out to evaluate the effect of XNJ on HBMECs. XNJ could improve rat cerebral ischemic injury and OGD induced HBMECs apoptosis. In vivo and in vitro researches indicated that the mechanism might be relevant to the activation of PI3K/Akt/eNOS signaling.

## 1. Introduction

Stroke remains an important major health problem with high morbidity, high disability, and high mortality worldwide, with 87% being ischemic stroke [1, 2]. Cerebral ischemia/reperfusion (I/R) injury occurs after effective thrombolysis therapy of stroke and can cause more serious secondary damage to brain tissue. Hence, it is urgent for us to find the exact mechanisms as well as the effective therapeutic drug for cerebral I/R injury. The pathophysiological mechanisms of cerebral I/R injury are very complex [3] and include energy metabolism impairment, oxidative stress, glutamate/neurotoxin release, calcium-overload, inflammation, apoptosis [4], and autophagy [5]. To date, apoptosis has been shown to play a role in cerebral I/R injury [6–9]; inhibition of apoptosis is a potential therapeutic target in stroke patients.

Endothelial nitric oxide synthase (eNOS) is the predominant NOS isoform and responsible for most of the NO produced in vessel, a loss of which results in vascular disease and various pathophysiological consequences. In regulating eNOS activity, Ser1177 phosphorylation is identified as one of the most important factors among the specific sites. A study of cerebral ischemia in rat showed that the phosphorylation of eNOS (p-eNOS) at Ser1177 regulates cerebral blood flow, represses apoptosis, and ameliorates the ischemic injury [10]. The phosphatidylinositol kinase-3/serine threonine kinase (PI3K/Akt) signaling pathway takes part in mediating cell survival and plays a critical role in I/R injury in the brain, heart, and kidney [11]. In addition, it has been testified that PI3K/Akt is critical in cerebral protection and regulating apoptosis induced by ischemia and hypoxia in brain tissue [12–14]. Previous studies have demonstrated that upregulating PI3K/Akt could stimulate phosphorylation of eNOS in many diseases including erectile dysfunction, alcoholic liver injury, hypertension, and stroke [15–18]. Thus, targeting PI3K/Akt and eNOS signaling pathway draws great attention for development of novel therapeutic strategies for cerebral ischemia.

**Figure 1:**
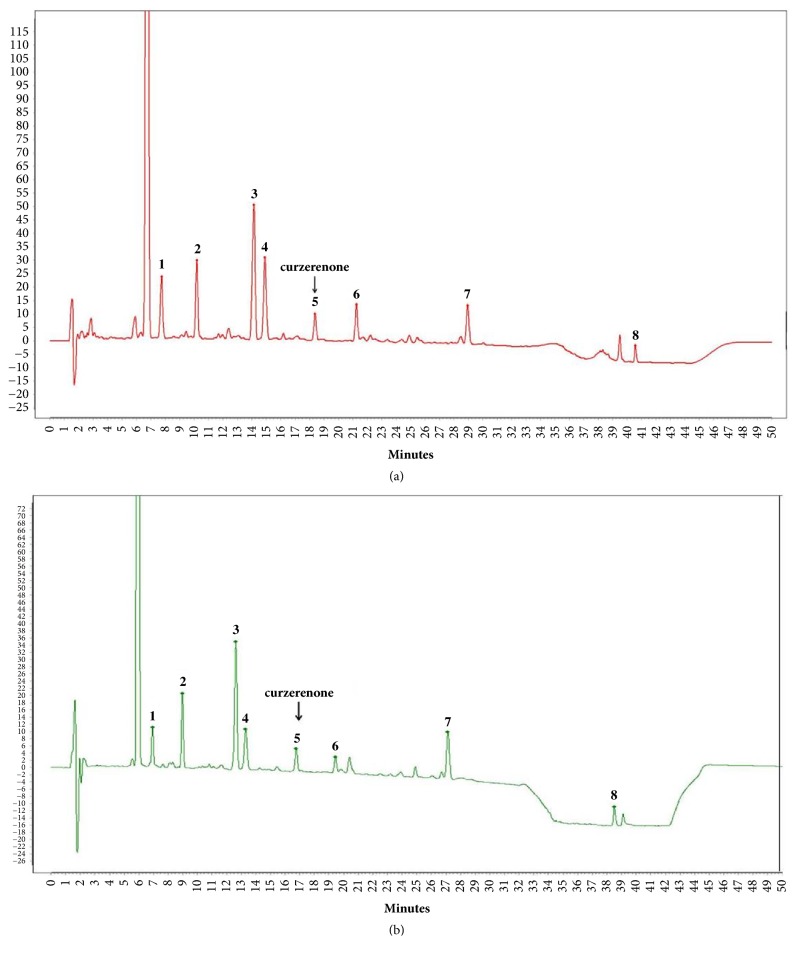
Typical HPLC chromatograms of curzerenone. (a) Standard curzerenone; (b) curzerenone in XNJ.

Xingnaojing (XNJ) is derived from An Gong Niu Huang Pill, a classic traditional Chinese medicine used to treat stroke in China. It predominantly contains Moschus (Moschus berezovskii F.; 7.5 g), Radix curcumae (Curcuma wenyujin Y.H. Chen & C. Ling, Zingiberaceae; 30 g), Borneolum (Blumea balsamifera DC, Compositae; 1 g), and Fructus gardenia (Gardenia jasminoides J. Ellis, Rubiaceae; 30 g) and is approved by the Chinese National Drug Administration [19]. Firstly XNJ was standardized using curzerenone, the fingerprint of XNJ (Figure 1(b)) containing 8 common peaks, and the HPLC retention time for curzerenone was consistent with that of standard curzerenone (Figure 1(a)). In addition, muscone and borneol could also be quality control substances, muscone should be no less than 0.1 mg, and borneol should be within 0.8~1.2mg mg per ml XNJ injection, which were detected by the gas chromatograph. Based on the above, the widely clinical use of XNJ is the quality-assurance. Clinical trials and pharmacological studies have proved that XNJ can relieve brain injury, promote functional recovery after stroke, and have neuroprotective effects in in vivo and in vitro models of stroke [19, 20]. Moreover, recent studies have indicated the neuroprotective effect of a herb pair of XNJ against ischemia stroke in rats [21]. However, the specific effects and mechanisms of XNJ on the cerebral ischemia reperfusion are not clear, which limits further clinical application. Here, we used the in vivo and in vitro models to further investigate whether the PI3K/AKT/eNOS pathway is involved in the effects of XNJ on cerebral I/R injury.

## 2. Materials and Methods

### 2.1. Reagents

XNJ was obtained from Henan Tiandi Pharmaceutical Co., Ltd. (Henan, China) with the Chinese Food and Drug Administration number z41020664. 2,3,5-Triphenyltetrazolium chloride (TTC) hematoxylin and eosin (H&E) staining kit was obtained from Sigma (St. Louis, MO). Antibodies against PI3K/Akt/eNOS, Bcl-2, Bax, caspase-3, actin, and GAPDH were purchased from Cell Signaling Technology (CST, California, USA). Nitric oxide (NO) assay kit (nitrate reductase method) was from NanJing JianCheng Bioengineering Institute (Nanjing, China). 4,6-Diamidino-2-phenylindole (DAPI), annexin V-FITC apoptosis detection kit, and JC-1 probe were purchased from Beyotime Institute of Biotechnology (Haimen, China). LY294002 was obtained from Selleckchem (Houston, USA). All other reagents were from common commercial sources.

### 2.2. Animal and Study Design

**Figure 2:**
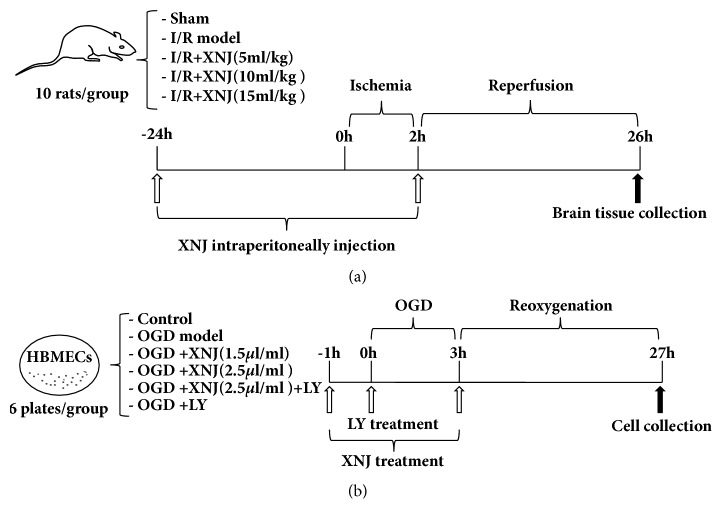
Study design of XNJ pretreatment and cerebral ischemia reperfusion injury exposure. (a) XNJ pretreatment and cerebral ischemia reperfusion (I/R) injury exposure in rats; (b) XNJ pretreatment and oxygen-glucose deprivation OGD exposure in human brain microvessel endothelial cells (HBMECs).

Sprague-Dawley rats (250–280 g) were purchased from Jilin University Animal Center for this research. All experimental animals were treated based on the National Institutes of Health Guide for the Care and Use of Laboratory Animals (NIH Publication No. 85-23, revised 1996). All experimental procedures were approved by the Animal Ethics Committee of Jilin University. Every effort was made to relieve the pain of animals. Rats (N = 50) were randomly divided into 5 groups of 10 rats each: Sham, I/R, I/R +XNJ (5ml/Kg), I/R +XNJ (10ml/Kg), and I/R +XNJ (15ml/Kg) (Figure 2(a)). The doses were selected according to that commonly used in clinical treatment. A modified middle cerebral artery occlusion (MCAO) was taken to accomplish focal cerebral ischemia on a heating lamp at 37°C [22]. Briefly, a 2 cm midline incision was performed on the neck, and the right external and internal carotid arteries were dissected carefully. A standard 4–0 nylon filament with a heat-blunted tip was inserted into the right internal carotid artery from the external carotid artery to block the origin of the MCA for 2 h. After 2 h of ischemia, the cerebral obstruction was withdrawn carefully to allow MCA reperfusion. Sham group received the same surgical operations except the occlusion/reperfusion. XNJ of three concentrations was injected intraperitoneally 24 h before the ischemia procedure and at set of reperfusions, respectively. Sham and I/R rats were given an equivalent volume of saline.

### 2.3. Estimation of the Neurological Score

After 24 h of reperfusion, neurological deficit scores were evaluated by an observer blinded to experimental groups as previously reported [23]. The scores were as follows: grade 0, normal (no apparent neurological deficits); grade 1, failure to entirely extend the contralateral forelimb; grade 2, circling continuously to the contralateral side but standard posture at rest; grade 3, falling to the injured side; grade 4, no spontaneous autonomic activity and a sluggish level of consciousness; grade 5, death. Rats with grades of 0 or 5 were withdrawn [24].

### 2.4. Estimation of Infarct Volume Was Assessed

24 h after I/R brain tissues were cut into 2 mm thick coronal sections (5 slices) using a brain slicer matrix and stained with 2% TTC (Sigma-Aldrich, Saint Louis, MO, USA) at 37°C for 30 min. The sections were photographed with a digital camera and the percentage of infarct area was quantified by Image J [13].

### 2.5. Cell Culture and Oxygen-Glucose Deprivation (OGD)

HBMECs (purchased from Chinese Academy of Medical Sciences) were cultured in DMEM supplemented with 10% fetal bovine serum (FBS, Gibco, USA). When 80–90% confluence was reached, the cells were digested with trypsin and then allocated randomly to six groups: control, OGD, OGD+XNJ (1.5*μ*l/ml), OGD+ XNJ (2.5*μ*l/ml), OGD+ XNJ (2.5*μ*l/ml)+LY294002, and OGD+ LY294002. OGD was constructed by culturing the cells with glucose and serum-free basic salt solution (BSS, 5 mmol·L-1 KCl, 120 mmol·L-1 NaCl, 1.2 mmol·L-1 CaCl_2_, 1.1mmol·L-1 KH_2_PO_4_, 20 mmol·L-1 Na2CO3, and 1.2 mmol·L-1 MgSO_4_). Next the plate was placed in an anaerobic chamber at 37°C with a controlled atmosphere of 5% CO_2_, 85% N_2_, and 10% H_2_ for 3 h. At last the BSS was placed with normal medium and the cells were put back to the normal incubator with 5% CO_2_ for 24 h to recover. Cells were subjected to XNJ or LY294002 (10*μ*M, Sigma-Aldrich) 1 h before OGD and at the onset of recovery (Figure 2(b)).

### 2.6. NO Assay

An NO2-/NO3-Assay Kit was taken to detect NO levels in brain tissues and supernatant of HBMECs by the Griess method according to the manufacturer's instruction.

### 2.7. HE Staining

After 24 h of reperfusion, the brains were fixed immediately with 4% paraformaldehyde overnight at 4°C and embedded in paraffin using an automatic tissue processor. Next 4 *μ*m thick tissue sections were cut using a microtome and stained with hematoxylin-eosin. The ipsilateral cerebral cortex of each group was observed under a light microscope to detect the morphology changes (Olympus).

### 2.8. Western Blot Analysis

RIPA lysis buffer was used to extract total protein from the ischemic penumbra of cerebral cortex and protein content was measured using a protein concentration assay kit (Beyotime Biotechnology, Beijing, China). Protein extracts (20 *μ*g/lane) were loaded on a SDS-PAGE gel and transferred electrophoretically to polyvinylidene fluoride membranes. After blocking for 1 h at room temperature in a fresh blocking buffer (containing 5% skim milk in 0.1% Tween-20 in Tris-buffered saline, pH 7.4), membranes were incubated with anti-eNOS, anti-PI3K/AKT, anti-Bcl2, anti-Bax, anti-caspase-3, anti-*β*-actin, or anti-GAPDH antibodies overnight at 4°C. After incubation with secondary antibody for 1 h at room temperature, membranes were exposed to ECL to visualize graphs and then quantified by Image J.

### 2.9. Immunofluorescent Assay and Nuclear Morphology Determination

HBMECs were seeded on a six-well plate and exposed to experimental procedures. 4% paraformaldehyde was used to fix cells and 0.5% Triton X-100 was used to permeabilize cells. For immunofluorescence, cells were blocked with 5% bovine serum albumin; first antibody specific to p-eNOS (1:100) was added to cells at 4°C overnight. FITC-labeled secondary antibody was incubated in dark place for 1 h. For nuclear morphology determination, the cells were stained with DAPI away from light for 5 min. A fluorescence microscope was used to inspect and record positive staining (Olympus).

### 2.10. Flow Cytometry

Annexin V-FITC apoptosis detection kit was used to measure the percentage of apoptotic cells. Concisely, after being subjected to the necessary experimental treatments, cells were digested with trypsin, blown with the original medium, and collected by centrifugation. Then cells were washed two times by PBS and resuspended by 210 *μ*L binding buffer (195 *μ*L annexin V-FITC, 5 *μ*L annexin FITC, and 10 *μ*L PI) and fixed for 10 min away from light. A flow cytometer was used to analyze the ratio of apoptotic cells.

### 2.11. Mitochondrial Membrane Potential Assay

JC-1 probe was taken to detect mitochondrial depolarization. Firstly, cells in 6-well plates were exposed to predesigned experimental conditions; then JC-1 staining solution (10*μ*g/mL) was added to cells and reacted at 37°C for 20 min. At last, cells were surveyed under a fluorescence microscope (Olympus) at 488 nm for green and red fluorescence. The relative value of green/red fluorescence intensity analyzed by Image J indicated mitochondrial membrane potential, and an enhancement in this ratio revealed mitochondrial depolarization.

### 2.12. Statistics

All the data were expressed as a mean ±standard derivation (SD). The difference between groups analysis was performed with one-way ANOVA followed by Dunnett's test or Student's t-test.* P* value<0.05 indicates the statistically significant difference. All the experiments were repeated at least three times.

## 3. Results

### 3.1. XNJ Ameliorated Cerebral I/R Injury in Rats

**Figure 3:**
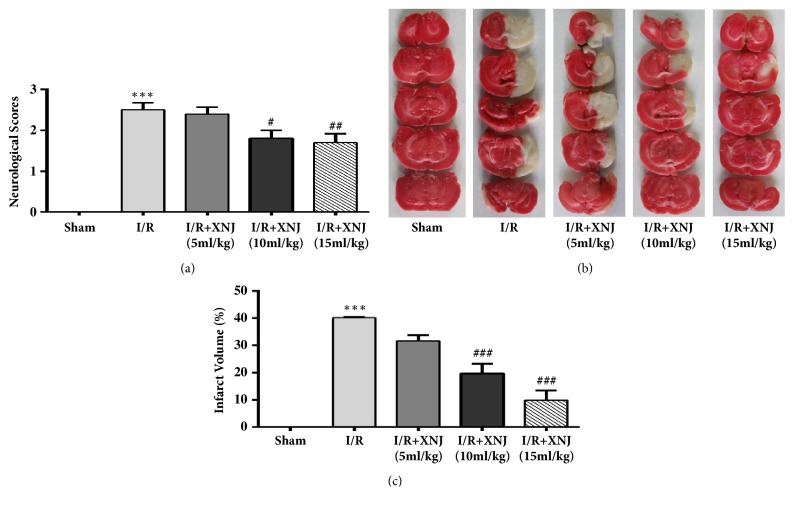
XNJ reduced cerebral ischemia reperfusion injury. (a) Neurologic evaluation. (b) Representative images of coronal brain sections stained with 2,3,5-triphenyltetrazolium chloride. (c) Statistical graph of cerebral infarct size in brains. The percentage of the opposite hemisphere area was used to express the volume of infarction. SD rats were intraperitoneally injected with saline (Sham group and I/R group) or 5 ml/kg, 10 ml/kg, 15 ml/kg XNJ 24 h before the ischemic injury and onset of reperfusion, respectively. Rats underwent 2 h of middle cerebral artery occlusion followed by 24 h of reperfusion. Data were expressed as means ± SD (n = 5-10 separated experiments). *∗∗∗p* < 0.001 vs. sham-operated group; #* p* < 0.05 vs. I/R group; ##*p* < 0.01 vs. I/R group; ###*p* < 0.001 vs. I/R group.

To evaluate the protective effects of XNJ against cerebral I/R damage, we examined neurological scores and infarct size 24 h after reperfusion in cerebral I/R injury rats. Neurological deficit scores of I/R group were higher in comparison with that of sham group, indicating I/R could lead to neurological function injury and the rat cerebral I/R model was successfully constructed. XNJ treatment prominently decreased the scores of neurological deficits compared with cerebral I/R group (Figure 3(a)). Similarly, TTC staining certified that the average cerebral infarct volume in XNJ group was significantly smaller than that in the I/R group (Figures 3(b) and 3(c)). The observed reduction in neurological deficit scores and infarct size suggested that XNJ might provide neuroprotection in cerebral I/R injury rats.

### 3.2. XNJ Prevented Morphology Change and Apoptosis in Rats

**Figure 4:**
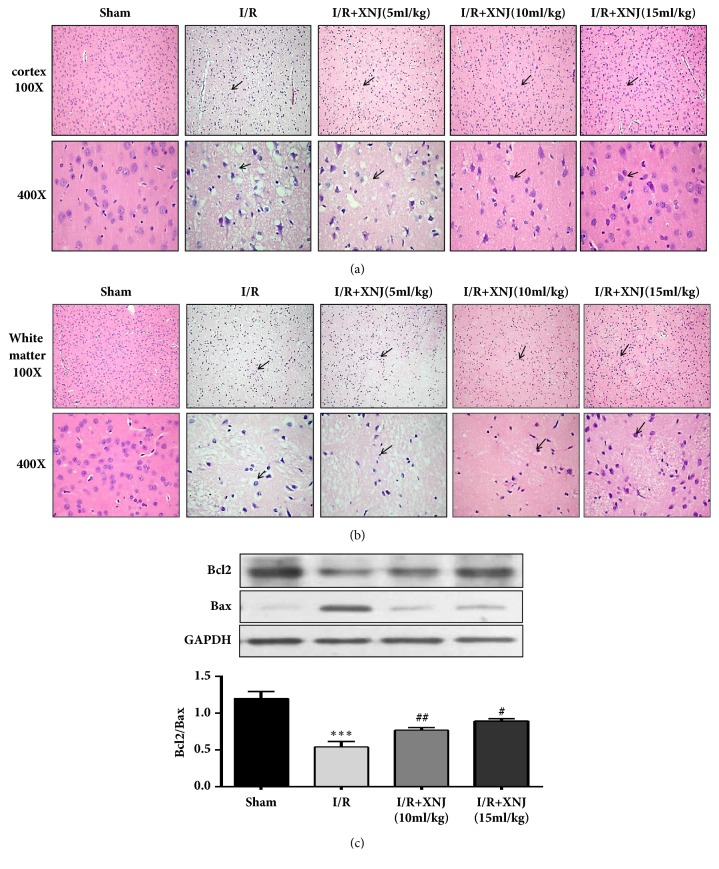
Effects of XNJ on histopathology and apoptosis. (a) H&E-stained cerebral cortex of I/R brain after 24 h of reperfusion (100× and 400×). (b) H&E-stained cerebral white matter of I/R brain after 24 h of reperfusion (100× and 400×) (scale bar = 50 *μ*m). The black arrow represents the pyknotic nucleus. (c) The ratio of Bcl2/Bax. Data were expressed as means ± SD (n = 5). *∗∗∗p* < 0.001 vs. sham group; #* p* < 0.05 vs. I/R group; ##*p* < 0.01 vs. I/R group.

To further explore the protective effects of XNJ against I/R brain injury, the morphology changes were observed by hematoxylin and eosin (H&E) staining after 24 h of reperfusion. At the cerebral cortex, the neuronal cells became a pyknotic nucleus (black arrow) and vacuole around the nucleus in the I/R group. The XNJ groups attenuated the neuronal impairments (Figure 4(a)). Consistently, leukoaraiosis appeared in the I/R group, which was alleviated by XNJ treatment (Figure 4(b)). No morphological changes in the cortex and white matter were observed in the sham group. To inspect the neuroprotective effects of XNJ against I/R via relief of apoptosis, western blotting was used to detect the expression of antiapoptosis protein Bcl2 and proapoptosis protein Bax in the penumbra area of the brain tissue. I/R group severely decreased the ratio of Bcl2/Bax, which was partly reversed by XNJ (Figure 4(c)).

### 3.3. XNJ Pretreatment Enhanced PI3K/Akt/eNOS Phosphorylation and NO Production in I/R Rat Brain Tissue

**Figure 5:**
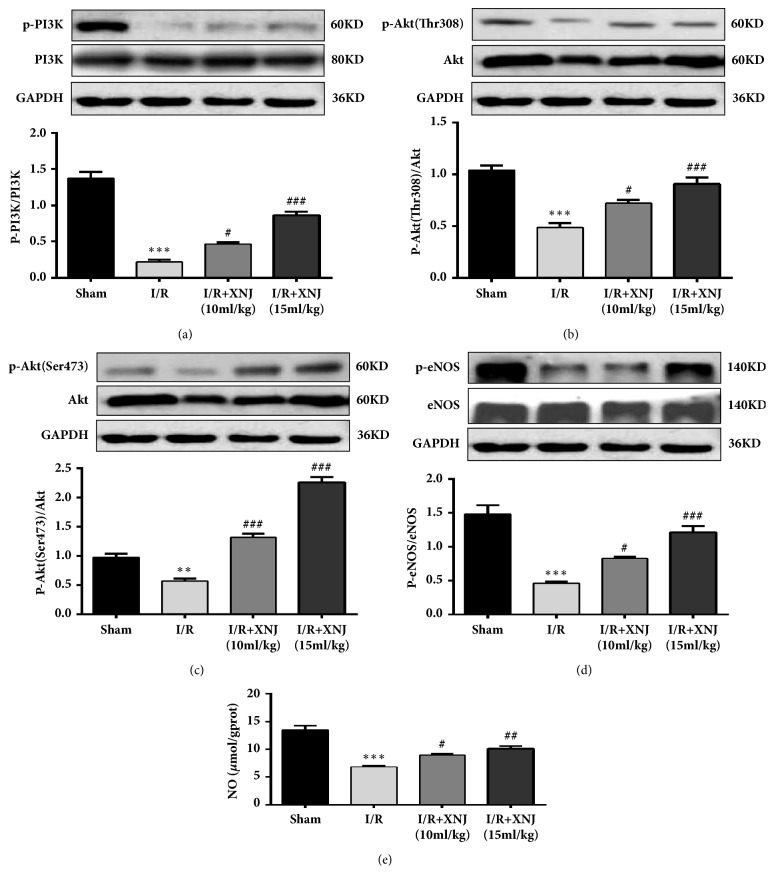
Effects of XNJ on the phosphorylations of PI3K (a), Akt308 (b), Akt473 (c), eNOS (d), and NO level (e) in the infarction of rat brain tissues. PI3K, phosphorylated PI3K, Akt, phosphorylated Akt at Thr308 [p-Akt (Thr308)] and Ser473 [p-Akt (Ser473)], eNOS, and phosphorylated eNOS were detected by western blot. The column diagrams show value of p-PI3K relative to that of PI3K, the value of p-Akt relative to that of Akt, and the value of p-eNOS relative to that of eNOS, respectively. NO level was measured by NO kit. Data were expressed as means ± SD (n = 5). *∗∗∗p* < 0.001 vs. sham group; #* p* < 0.05 vs. I/R group; ##*p* < 0.01 vs. I/R group; ###*p* < 0.001 vs. I/R group.

Mounting evidence showed that the activation of the PI3K/Akt signaling pathway induces protection against cerebral I/R and NO production increment might be related to the induction of eNOS phosphorylation. To estimate the effects of XNJ on** I/R rat brain**, we measured the effect of XNJ on the activation of PI3K/Akt/eNOS signaling and NO production in the brain tissues. Since there were statistically significant improvements in neurological function and infarct volume at 10 ml/kg and 15 ml/kg XNJ, the rest of the study was conducted using these two doses. The result indicated XNJ treatment significantly increased the levels of phospho-PI3K/Akt in the brain tissues of compared with untreated I/R group (Figures 5(a), 5(b), and 5(c)). Similarly, cerebral I/R decreased the levels of phospho-eNOS compared with sham control, which was reversed by XNJ treatment (Figure 5(d)).  Figure 5(e) showed that XNJ administration markedly increased the levels of NO compared with I/R group, which was consistent with the above results.

### 3.4. XNJ Induced eNOS Phosphorylation and NO Level Upregulation via PI3K/Akt Signaling in HBMECs Subjected to OGD

**Figure 6:**
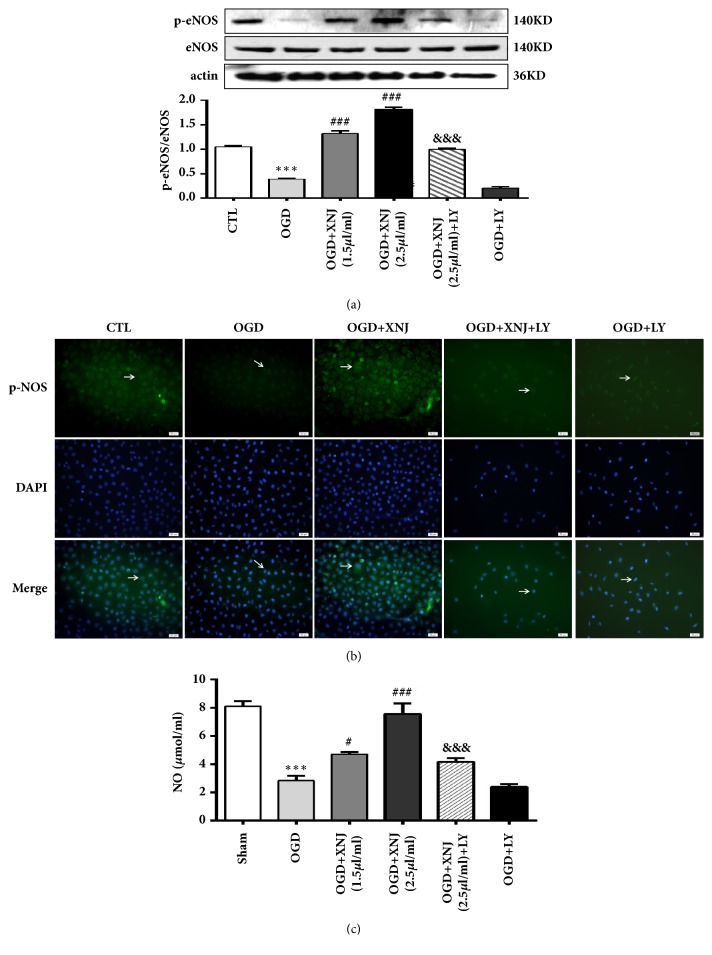
XNJ induced endothelial NO synthase (eNOS) phosphorylations and nitric oxide (NO) enhancement in HBMECs after exposure to “OGD”. HBMECs were treated with XNJ (1.5*μ*l/ml, 2.5*μ*l/ml) and received 3 h OGD followed by 24 h recovery. LY294002 (10*μ*M) was added to XNJ (2.5*μ*l/ml) group. (a) The protein expression of phosphorylated eNOS (p-eNOS) at Ser-1177 and eNOS was analyzed by western blot; (b) p-eNOS was upregulated in the HBMECs exposed to OGD pretreated with XNJ (2.5*μ*l/ml) by immunofluorescent staining. Positive stainings as shown in green. Scale bars: 50 *μ*m. (c)NO production in supernatant was assayed by Griess method. LY294002 (10*μ*M) pretreatment alleviated the XNJ induced eNOS phosphorylation and NO production. Data were expressed as means ± SD (n = 4). *∗∗∗ p* < 0.001 vs. CTL; #*p* < 0.05 vs. OGD group; ##*p* < 0.01 vs. OGD group; ###*p* < 0.001 vs. OGD group; &* p* < 0.05 vs. OGD +XNJ (2.5*μ*l/ml) group; &&* p* < 0.01 vs. OGD +XNJ (2.5*μ*l/ml) group.

To explain the mechanisms underlying the enhancement of eNOS phosphorylation after XNJ treatment in HBMECs exposed to “OGD”, we used LY294002, a specific inhibitor for PI3K/Akt, to test the involvement of PI3K/Akt in this response. XNJ reversed phosphorylations eNOS (Figures 6(a) and 6(b)) and NO level downregulation (Figure 6(c)) induced by “OGD”, which were significantly attenuated by LY294002. These results implied an essential role of PI3K/Akt signaling in XNJ-mediated eNOS activation in OGD cultured HBMECs

### 3.5. PI3K/Akt/eNOS Signaling Pathway Is Crucial for the Protective Effect of XNJ against HBMECs Apoptosis

**Figure 7:**
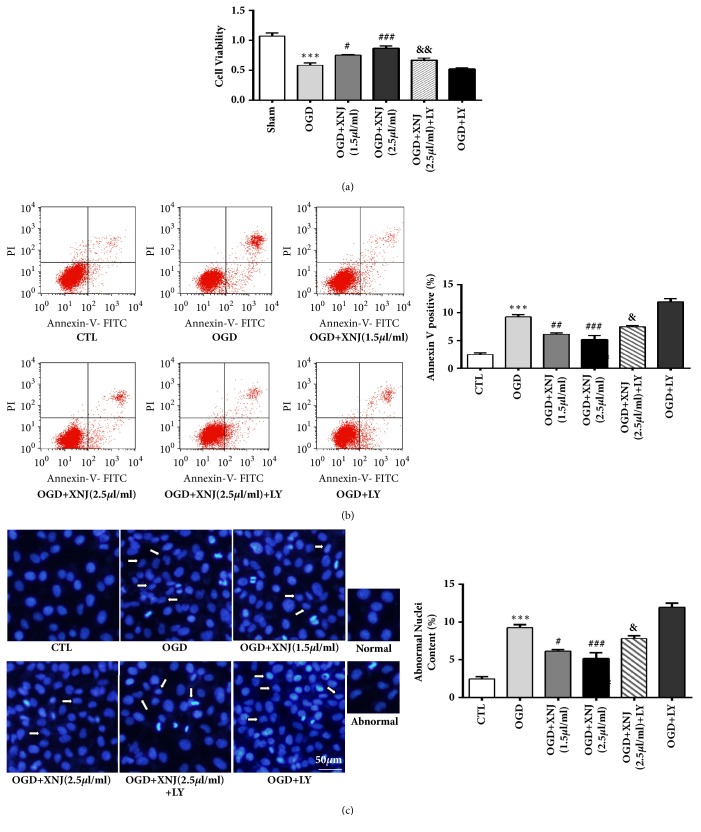
PI3K/AKT/eNOS regulated the remission of XNJ on apoptosis in HBMECs. (a) Cells viability was measured by MTT. (b) Apoptotic cells in each group were detected in a flow cytometer. The quantification of annexin V-positive cells (apoptotic cells) was presented in the histogram. (c) Representative fluorescent photographs of cells stained with DAPI and the quantitative statistical charts of the abnormal nuclei ratio. White arrow labeled abnormal (crenation, shrinkage, and fractionation) and normal nuclei. Scale bar = 50 *μ*m. Data were expressed as means ± SD (n = 5). *∗∗∗ p* < 0.001 vs. CTL; #*p* < 0.05 vs. OGD group; ###*p* < 0.001 vs. OGD group; &&&* p* < 0.001 vs. OGD +XNJ (2.5*μ*l/ml) group.

**Figure 8:**
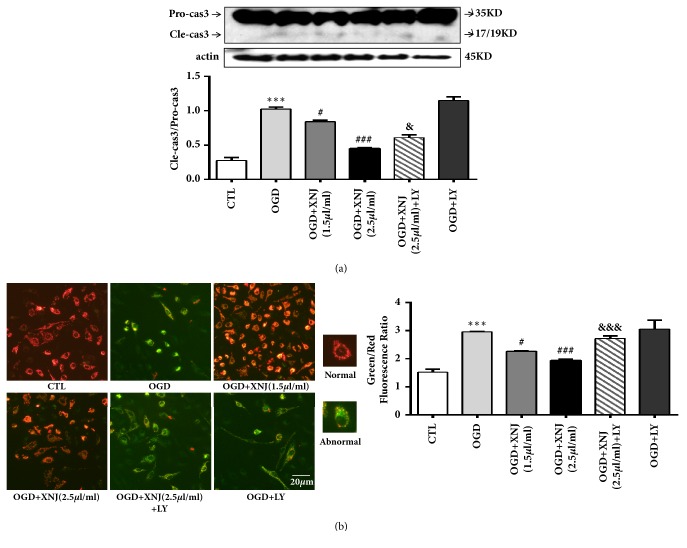
XNJ alleviated HBMECs apoptosis via regulating caspase activity and mitochondrial potential. (a) The protein expression of procaspase-3 and cleaved caspase-3 was analyzed by western blot. The ratio of cleaved caspase-3/procaspase-3 showed caspase-3 activation. (b) Mitochondrial membrane potential was measured by JC-1 probe. When the mitochondrial membrane potential is high, cells glow with strong red fluorescence; in opposite, cells glow with strong green fluorescence. The relative value of green/red fluorescence indicated the ratio of mitochondrial depolarization in cells. All values came from individual images of each group. Scale bar = 20 *μ*m. Data were expressed as means ± SD (n = 4). *∗∗∗ p* < 0.001 vs. CTL; #*p* < 0.05 vs. OGD group; ###*p* < 0.001 vs. OGD group; &* p* < 0.05 vs. OGD +XNJ (2.5*μ*l/ml) group; &&&* p* < 0.001 vs. OGD +XNJ (2.5*μ*l/ml) group.

As PI3K/Akt/eNOS is potential in regulating apoptosis, we purposed to investigate the role of PI3K/Akt/eNOS in XNJ-mediated antiapoptotic effect in OGD cultured HBMECs. Firstly, cell viability was measured by MTT and apoptotic ratio was detected by flow cytometer analysis; the results showed that XNJ significantly improved the decreased cell viability and increased apoptotic ratio induced by OGD, which was partly reversed by PI3K/Akt inhibitor LY294002 (Figures 7(a) and 7(b)). Next, we detected nucleus morphology using DAPI staining and the ratio of abnormal cells was quantitatively analyzed (Figure 7(c)). The normal nuclei of cells in the control group were round or oval, with evenly distributed karyoplasm and clear boundaries. However, in the OGD group, apoptosis was observed, as shown by nuclear condensation, margination, and fragmentation, and some nuclei were lobular. XNJ treatment reduced nuclear deformation induced by OGD, which was partly reversed by inhibiting PI3K/Akt. The nuclei stained with DAPI showed irregular small pieces (arrow). During apoptosis, caspase family was crucial in the apoptotic proteolytic events. Caspase-3 was always regarded as apoptotic indicator; given that caspase-3 is activated by the cleavage of its precursor, procaspase-3, we measured the effect of XNJ and OGD on caspase-3 activity by detecting the expression of procaspase-3 and cleaved caspase-3. Western blot results showed that XNJ weakened the enhanced activity of caspase-3 induced by “OGD”, while this effect was whittled when PI3K/AKT was inhibited (Figure 8(a)). Caspase activity is a contributor to mitochondrial damage. The mitochondrial damage was marked by loss of mitochondrial membrane potential (△*Ψm*). Hence, to further explore the effect of XNJ on apoptosis, JC-1 probe was applied to detect mitochondrial membrane potential. JC-1 probe gathers in the intact mitochondria matrix of normal cells, producing red fluorescence (high △*Ψm*), and is distributed in the injured mitochondria of apoptotic cells, producing green fluorescence (low △*Ψm*). The result in Figure 8(b) showed that OGD elevated the ratio of red (high △*Ψm*) to green (low △*Ψm*), indicating that mitochondrial membrane potential decreased and mitochondria were injured. XNJ reversed the loss of mitochondrial membrane potential induced by OGD, which was abolished to some extent by LY294002. The findings above declared that PI3K/Akt/eNOS signaling pathway was an important mediator in regulating the protective effect of XNJ against apoptosis induced by OGD in HBMECs.

## 4. Discussion

**Figure 9:**
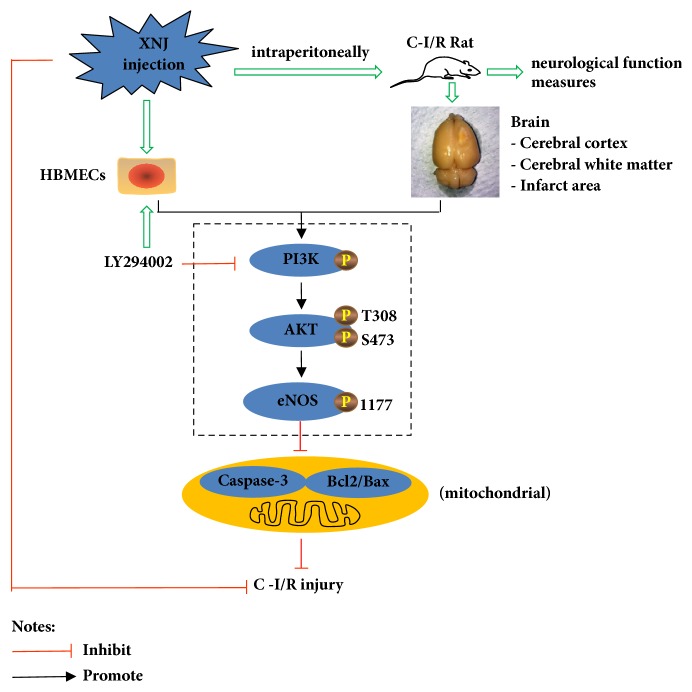
Schematic of XNJ induced protection against cerebral ischemia reperfusion injury in rats and HBMECs, illustrating potential mechanisms related to ameliorating cerebral ischemia reperfusion injury.

Although previous studies suggested that XNJ was protective in stroke, the effects and mechanisms have yet to be investigated. The present study provided novel data that XNJ significantly improved neurological function, brain infarction, and morphological changes in vivo, as well as improving cell viability and apoptosis in vitro. These protective effects might relate to eNOS phosphorylation via the PI3K/Akt signaling pathway (Figure 9).

Apoptosis was known to be involved in cerebral I/R injury. The antiapoptotic protein Bcl-2 and proapoptotic protein Bax localized to the mitochondria in response to numerous apoptotic stimuli and further activated the caspase cascade [25]. Therefore Bcl-2 and Bax are important in a number of apoptotic signaling pathways. It has been shown that cerebral I/R injury may expedite physiological apoptosis by decreasing the expression of Bcl2, elevating Bax [26]. The results from the present study supported this finding and clarified that pretreatment with XNJ reduced these changes in rat cerebral tissue. Endothelial NO synthase (eNOS) has been proved to play a critical role in proliferation and apoptosis by suppressing the proapoptotic proteins [27, 28]. NO produced by eNOS is considered to be a protective event against brain I/R injury [29–31]. As a critical modulator of eNOS activity and the outcome of ischemic injury, the eNOS phosphorylation at serine residue 1177 is usually used to evaluate cerebrovascular function. Furthermore, modulation of the eNOS S1179 phosphorylation site affects cerebral blood flow in vivo and influences stroke size following cerebral ischemia. Though eNOS activity is regulated by multiple mechanisms, PI3K/Akt are key enzymes controlling eNOS phosphorylation; blocking of PI3K/Akt can partially inhibit eNOS activity [32]. Importantly, it has been demonstrated that phosphorylation of PI3K/Akt/eNOS was diminished in the brain tissue of cerebral I/R rats compared with that in sham rats [33]. In this study, we established a rat model of transient focal cerebral ischemia because most attacks of cerebral ischemia are focal in clinic. Consistently, we found that P-PI3K, p-Akt at Ser437 and Thr308, and p-eNOS at Ser1177 in brain tissue were much lower after ischemia reperfusion than sham group; anyhow, all of them are increased by XNJ pretreatment. Clearly, XNJ increased the ratio of Bcl-2/Bax; in the meantime, it enhanced PI3K/Akt/eNOS phosphorylation. These findings indicated PI3K/Akt/eNOS phosphorylation may contribute to the protection of XNJ against C I/R.

As the first target of stroke, HBMECs play a key role in brain vascular repair and maintenance, and their function is impeded in stroke [34]. Reduction of the apoptosis of HBMECs after reperfusion is significant for the treatment of cerebral ischemia reperfusion injury. Hence, we established an in vitro injury model using OGD cultured HBMECs to mimic cerebral I/R injury for further studying the mechanism of XNJ alleviating I/R cerebral injury and what role PI3K/Akt/eNOS pathway plays in this process. XNJ administration has been testified to restore PC12 function and ameliorates neuroapoptosis in the infant rat striatum. However, little is known about the effect of XNJ on HBMECs. The present study shows that XNJ has a protective effect on OGD cultured HBMECs. Activation of PI3K/Akt signaling has been reported to protect cells from apoptosis induced by OGD [35–37] and similar results were shown in our study. In cultured HBMECs, we found that the protective effects of XNJ against apoptosis induced by OGD were partly reversed by LY294002. What is more, repression of eNOS activity and NO production induced by OGD was counteracted by XNJ treatment via PI3K/Akt-mediated eNOS phosphorylation at Ser1177. Activated caspase-3 and decreased mitochondrial membrane potential are likely to be involved in HBMECs apoptosis during GOD [38, 39]. Caspase-3 is a cysteine protease that is involved in mediating cell apoptosis. It results in cell shrinkage, DNA degradation, and the formation of apoptotic bodies [40]. In the present study, we found XNJ decreased caspase-3 activity and cell shrinkage and alleviated mitochondrial depolarization induced by OGD in HBMECs, which was reversed by LY294002. That is, PI3K/Akt/eNOS pathway was essential for XNJ protecting against HBMECs apoptosis.

In conclusion, XNJ ameliorates rat cerebral I/R injury and protects against OGD induced HBMECs apoptosis. These effects are possibly associated with regulating the phosphorylation levels of the PI3K/Akt/eNOS signaling pathway.

## Data Availability

The data used to support the findings of this study are included within the article.
